# Supplementary figures and images for: PCV2 targets cGAS to inhibit type I interferon induction to promote other DNA virus infection
Source: PLoS Pathog. 2021 Sep 20;17(9):e1009940. doi: 10.1371/journal.ppat.1009940 (PMC8483418; doi:10.1371/journal.ppat.1009940)

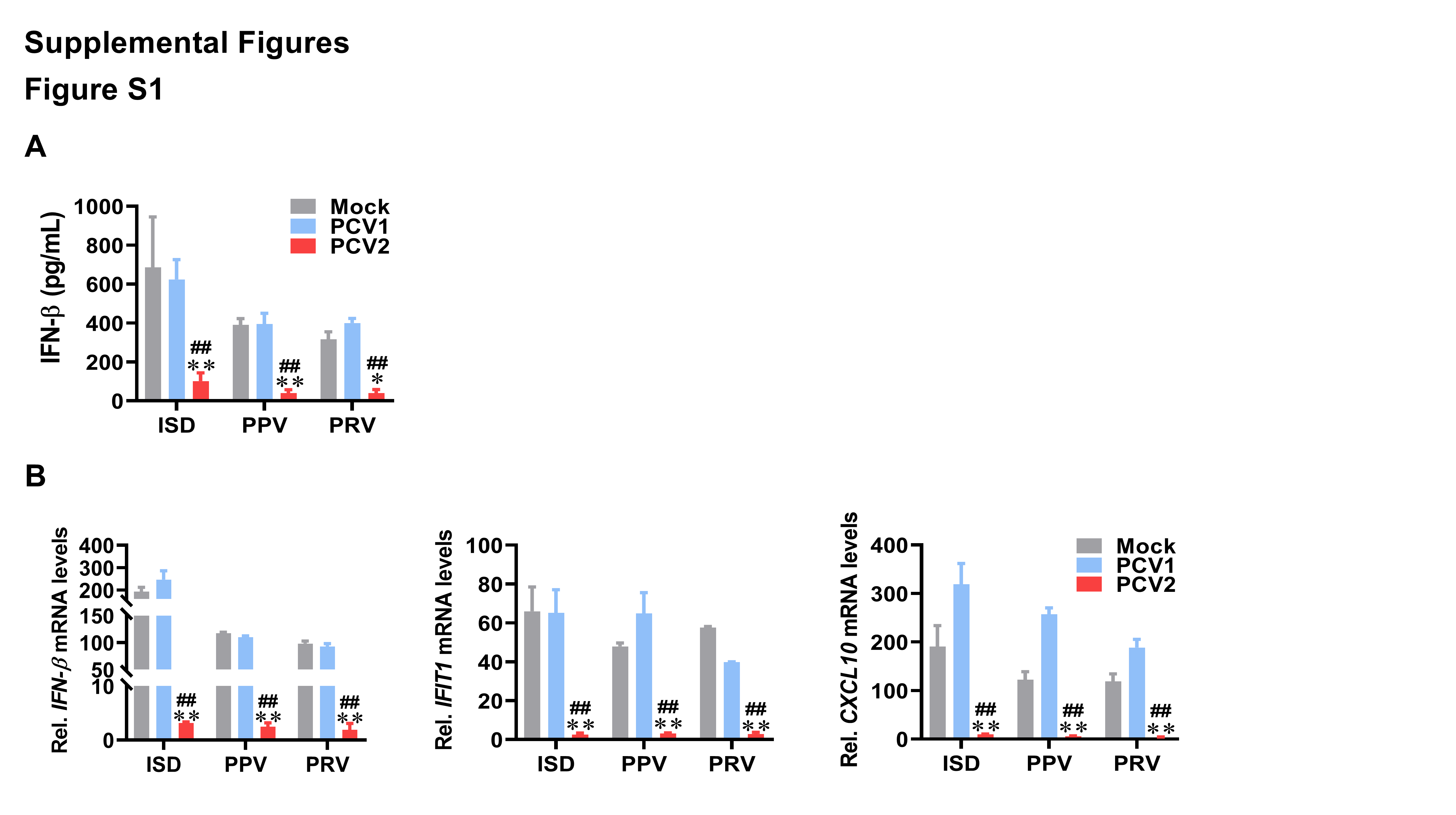

Supplement: S1 Fig — (A, B) PCV2 inhibits PPV- or PRV-induced IFN-β production and response. The PK-15 cells were infected by PCV1 (MOI = 5), PCV2 (MOI = 5), or Mock (same volume of medium) for 48 h, respectively, and then challenged with 1 MOI PPV, PRV or ISD for another 6 h. The supernatant IFN-β levels were measured by ELISA (A). IFN-β, IFIT1, and CXCL10 mRNA levels were determined by Q-PCR (B). * P < 0.05, ** P < 0.01 (compared with mock infection); # P < 0.05, ## P < 0.01 (compared with PCV1 infection). (TIF) [file ppat.1009940.s001.tif]

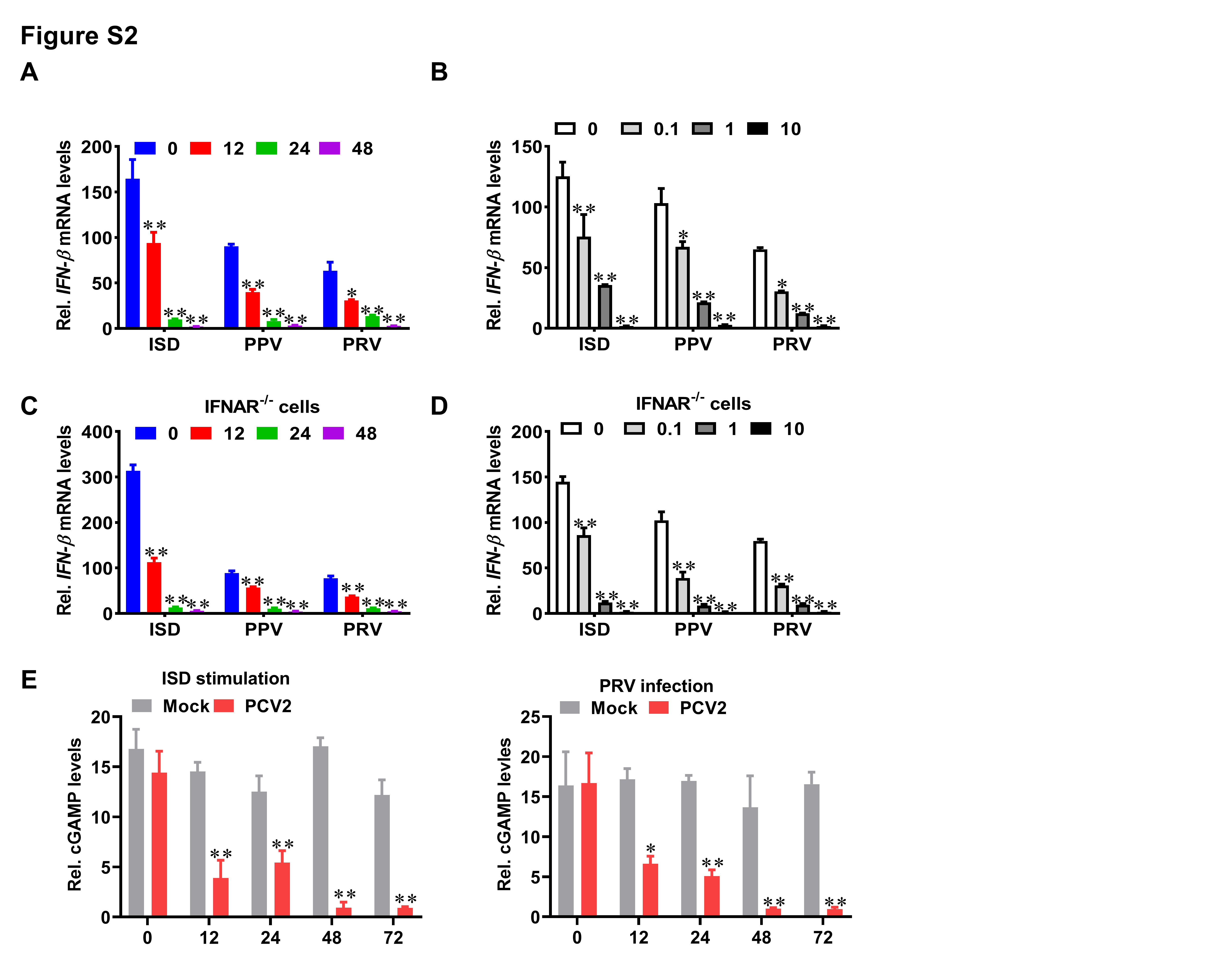

Supplement: S2 Fig — (A-D) The wild type PK-15 cells (A, B) and IFNAR-/- PK-15 cells (C, D) were infected with PCV2 (MOI = 5) for the indicated time (A, C), or infected with different doses (0.1, 1, 10 MOI) of PCV2 for 48 h (B, D), and then the relative IFN-β mRNA levels were determined by Q-PCR at 6 h following ISD stimulation or PPV or PRV infection. (E) PK-15 cells were infected with PCV2 (MOI = 5) for the indicated time, and then the relative cGAMP levels were determined at 6 h following ISD stimulation or PRV infection. * P < 0.05, ** P < 0.01, compared with infection at 0 h (A, C), 0 MOI PCV2 (B, D), or Mock infection (E). (TIF) [file ppat.1009940.s002.tif]

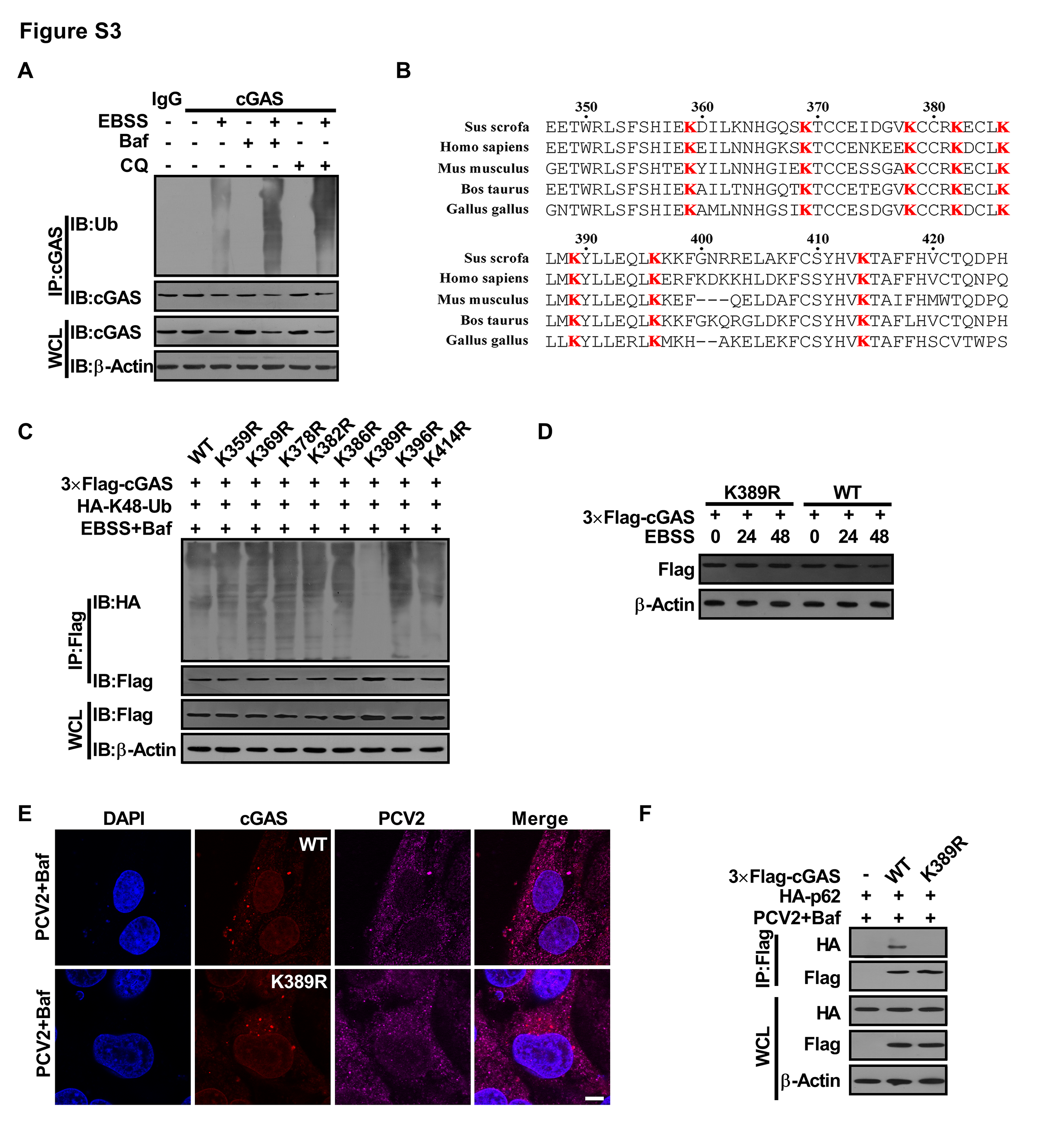

Supplement: S3 Fig — (A) EBSS treatment induces the poly-ubiquitination of cGAS. PK-15 cells were treated with EBSS along with or without Baf or CQ for 48 h. Cell lysates were analyzed by immunoprecipitated with anti-porcine cGAS antibody, and ubiquitinated cGAS proteins were immunoblotted using anti-ubiquitin antibodies. (B) Alignment of cGAS amino acid partly sequences. Highlighted amino acids indicate conserved lysine (K) of cGAS. (C) EBSS treatment promotes the K48-linked poly-ubiquitination of porcine cGAS at K389. PK-15 cells were transfected with different HA-Ub constructs as indicated, then were treated with EBSS along with Baf for 48 h. Cell lysates were immunoprecipitated with anti-Flag antibody and immunoblotted with anti-HA antibody. (D) K389R cGAS mutant degradation was abrogated in EBSS-treated cells. PK-15 cells were transfected with plasmids as indicated, then were treated with EBSS for the indicated time. cGAS levels were analyzed by western blotting. (E) Poly-ubiquitination of porcine cGAS at K389 is required for the interaction of cGAS with p62. The cGAS-/- PK-15 cells expressed Flag-cGAS, Flag-cGAS (K389R) were infected with PCV2 in the presence of Baf. The localization of porcine cGAS and PCV2 Cap protein was observed under confocal microscopy. Scale bar, 10 μm. (F) Co-immunoprecipitation experiment to test the affinity of WT and K389R cGAS to p62 in the cGAS-/- PK-15 cells that transfected with HA-p62 and 3×Flag-cGAS or 3×Flag-cGAS (K389R) constructs. (TIF) [file ppat.1009940.s003.tif]

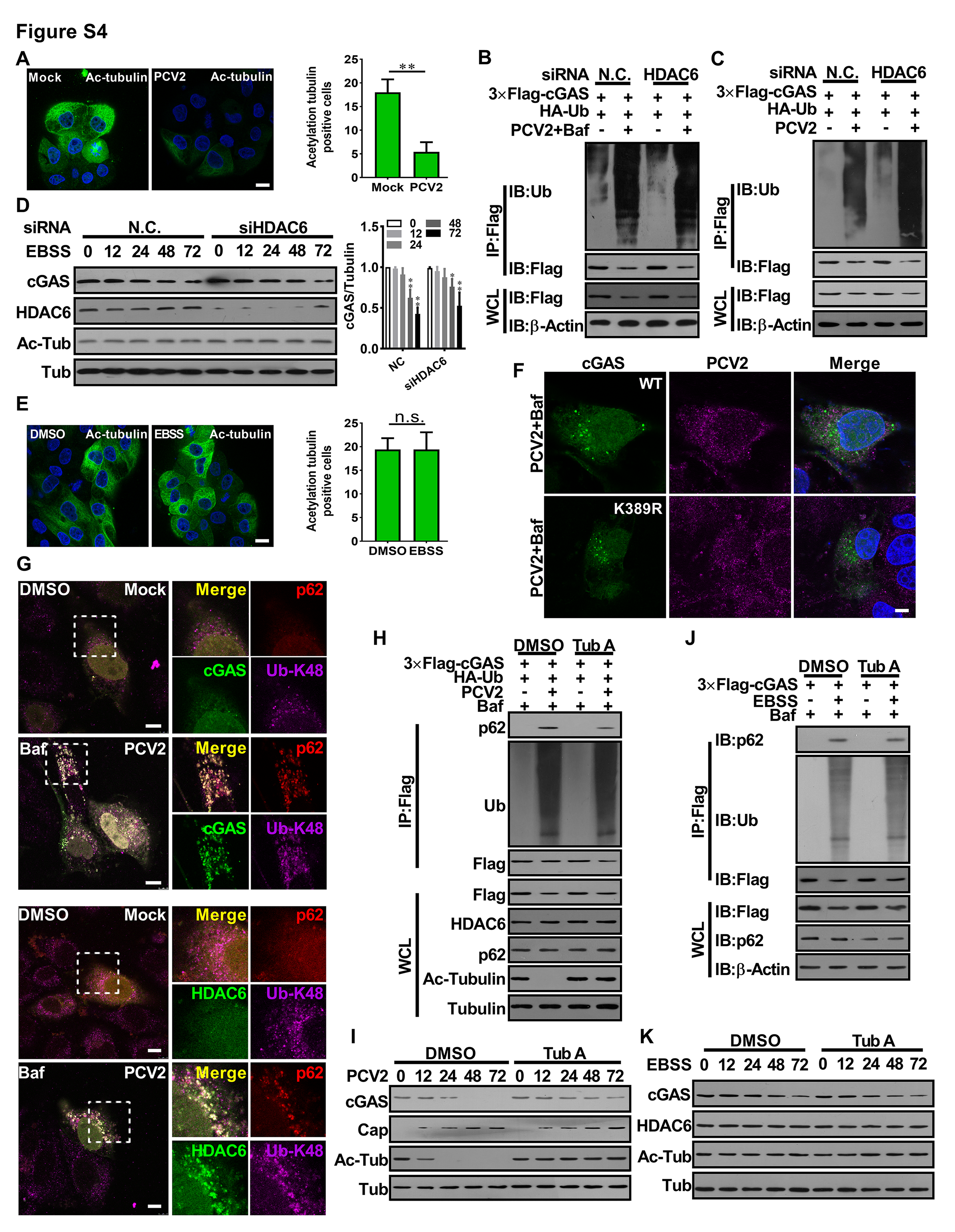

Supplement: S4 Fig — (A) Detection of the acetylated tubulin levels to determine the deacetylase activity of HDAC6 in PCV2 or mock infection cells. Statistical analysis of the Ac-tubulin levels in the indicated samples. Scale bar, 10 μm. ** P < 0.01. (B) PK-15 cells were transfected with HDAC6 specific siRNA (siHDAC6) or siRNA negative control (siN.C.) and other plasmids as indicated for 24 h. Then infected with PCV2 (MOI = 5) or mock in the presence of Baf for 48 h. The poly-ubiquitination levels and protein levels of cGAS were analyzed. (C) PK-15 cells were transfected with HDAC6 specific siRNA (siHDAC6) or siN.C. and other plasmids as indicated for 24 h. Then infected with PCV2 (MOI = 5) or mock in the absence of Baf for 48 h. The poly-ubiquitination levels and protein levels of cGAS were analyzed. (D) PK-15 cells were transfected with (siHDAC6) or siN.C. for 24 h, then treated with EBSS to detect the levels of porcine cGAS, HDAC6, and Ac-Tubulin at indicated times. (E) Detection of the acetylated tubulin levels to determine the deacetylase activity of HDAC6 in EBSS-treated or untreated cells. (F) The cGAS-/- PK-15 cells transfected with Flag-cGAS, Flag-cGAS (K389R) expression constructs were infected with PCV2 in the presence of Baf. The localization of porcine cGAS and PCV2 Cap protein was observed under confocal microscopy. Scale bar, 10 μm. (G) PK-15 cells were infected with PCV2 in the presence of Baf, then the colocalization of porcine cGAS, HDAC6, K48-Ub, and p62 were observed under confocal microscopy. Scale bar, 10 μm. (H) PK-15 cells transfected indicated plasmids were treated with Tub A for 6 h, then infected with PCV2 (MOI = 5) for another 48 h, and the interaction of ubiquitinated cGAS with p62 was analyzed. (I) PK-15 cells were pretreated with Tub A and infected with PCV2 (MOI = 5) for the indicated time, and then the levels of porcine cGAS, PCV2 capsid, and Ac-Tubulin were determined by western blotting. (J) PK-15 cells expressed Flag-cGAS were treated with EBSS for 48 [file ppat.1009940.s004.tif]

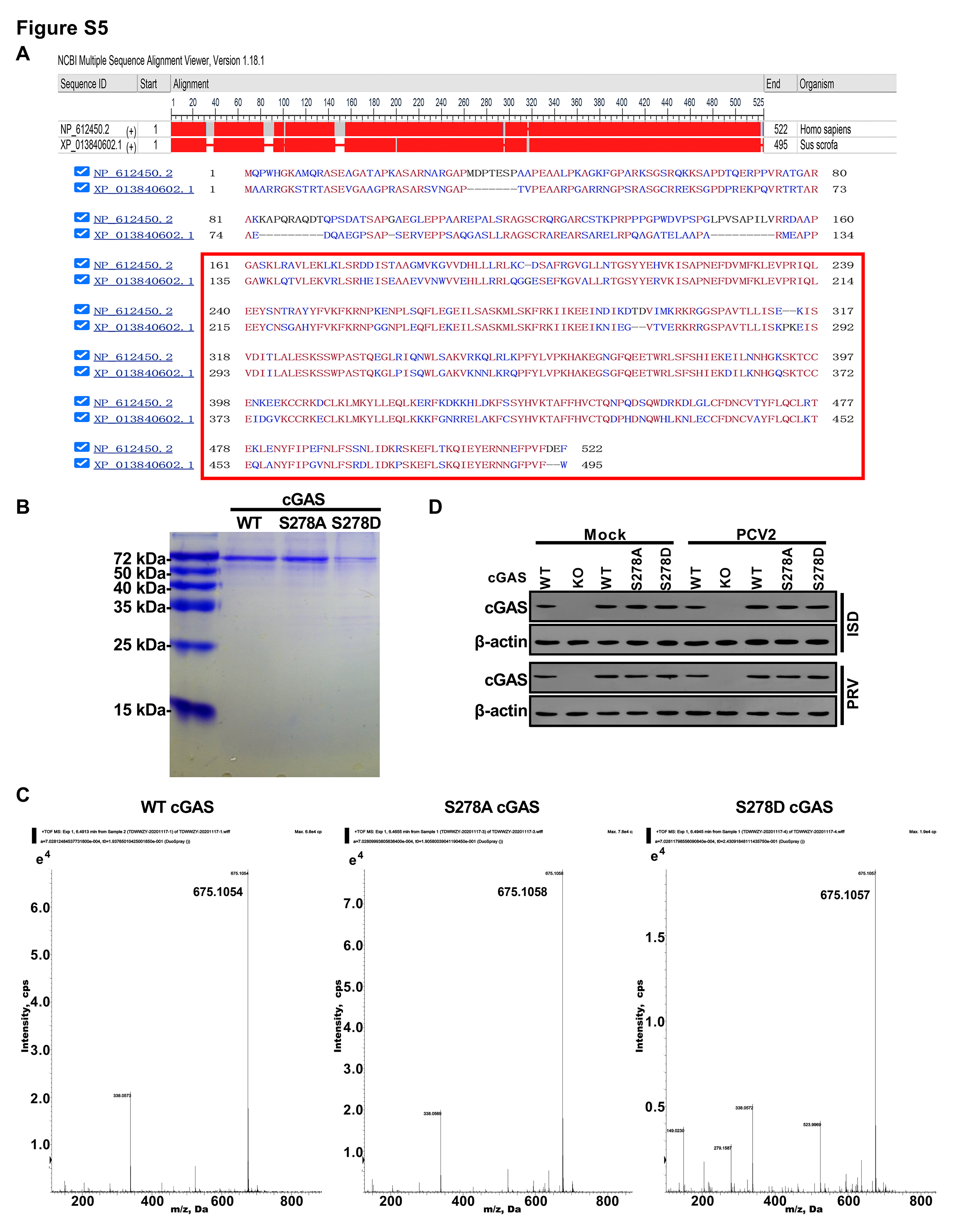

Supplement: S5 Fig — (A) Alignment of cGAS sequences between human and pig. (B) The purification of porcine cGAS WT, cGAS S278A, and cGAS S278D protein for in vitro enzymatic assay. Visualized by Coomassie brilliant blue staining. (C) LC-MS analysis of cGAMP production from an in vitro cGAMP synthesis assay. Small molecules were extracted from in vitro tubes for analysis of cGAMP isomers by tandem mass spectrometry. (D) The cGAS-/- PK-15 cells reconstituted with the WT cGAS, or cGAS mutant S278A, or cGAS mutant S278D were infected with mock or PCV2 in the presence of Baf for 12 h, and then these protein levels were detected by western blotting. (TIF) [file ppat.1009940.s005.tif]

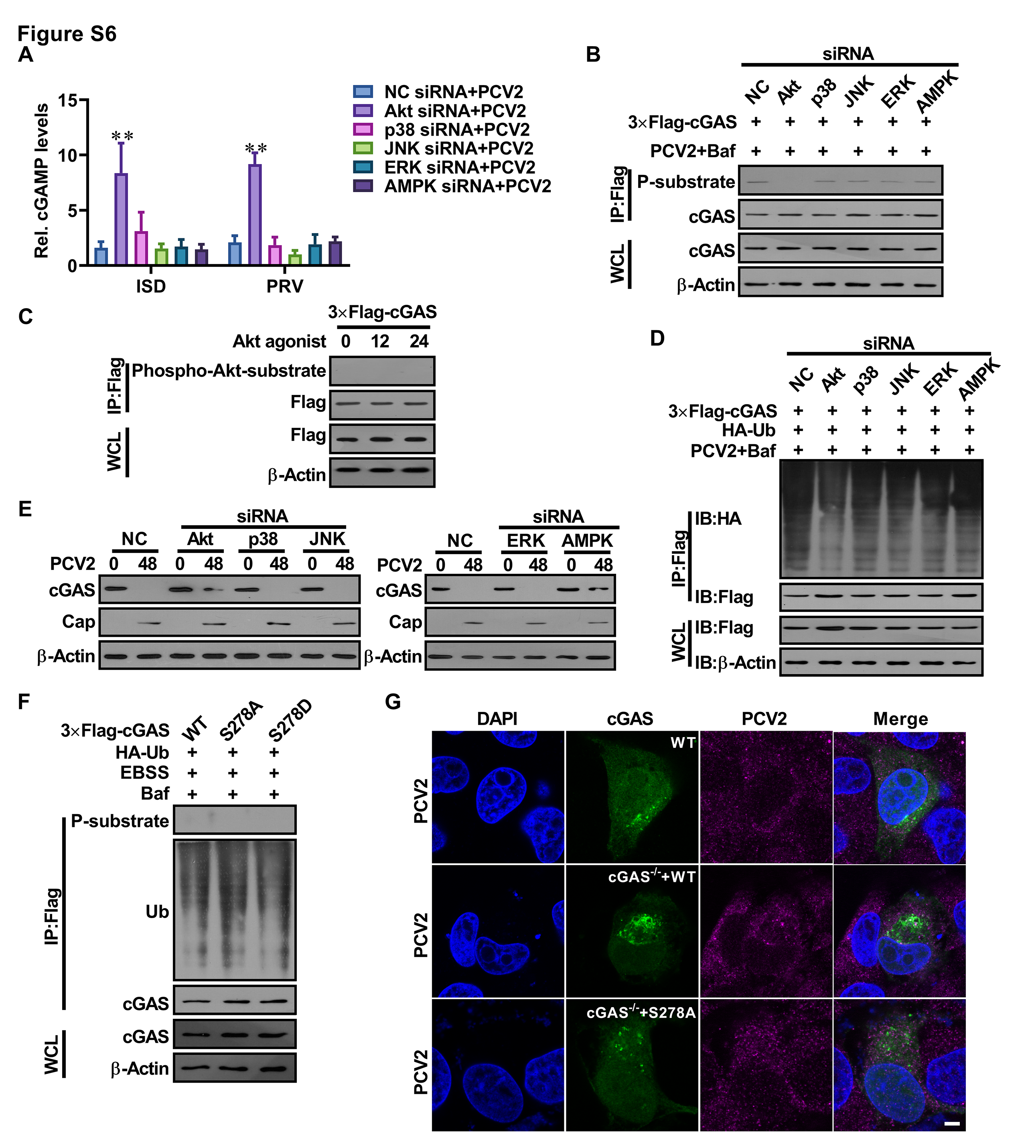

Supplement: S6 Fig — (A-B) PK-15 cells transfected with indicated siRNA were infected with PCV2 (MOI = 5) for 12 h, and then the relative cGAMP production levels were determined by report assay at 6 h following ISD stimulation or PRV infection (A); the phosphorylation level of cGAS at the S278 site was detected by western blotting (B). * P < 0.05, ** P < 0.01 (compared with siN.C.). (C) PK-15 cells were treated with Akt agonist (SC79) for indicated times, and the phosphorylation level of cGAS at the S278 site was detected by western blotting. (D-E) The phosphorylation of cGAS facilitates the ubiquitination and degradation of cGAS during PCV2 infection. PK-15 cells were transfected with indicated expression constructs and indicated siRNA, and then infected with PCV2 (MOI = 5) in the presence or absence of Baf to detect the poly-ubiquitination levels and protein levels of cGAS. (F) EBSS-induced cGAS ubiquitination is independent of the phosphorylation. The cGAS-/- PK-15 cells were reconstituted with the WT cGAS, S278A mutant, or S278D mutant, treated with EBSS to detect the poly-ubiquitination levels and phosphorylation levels of cGAS. (G) The wild-type PK-15 cells and cGAS-/- PK-15 cells were transfected with Flag-cGAS or Flag-cGAS (S278A) expression constructs, then infected with PCV2 to observe the localization of porcine cGAS and PCV2 Cap protein. Scale bar, 10 μm. (TIF) [file ppat.1009940.s006.tif]

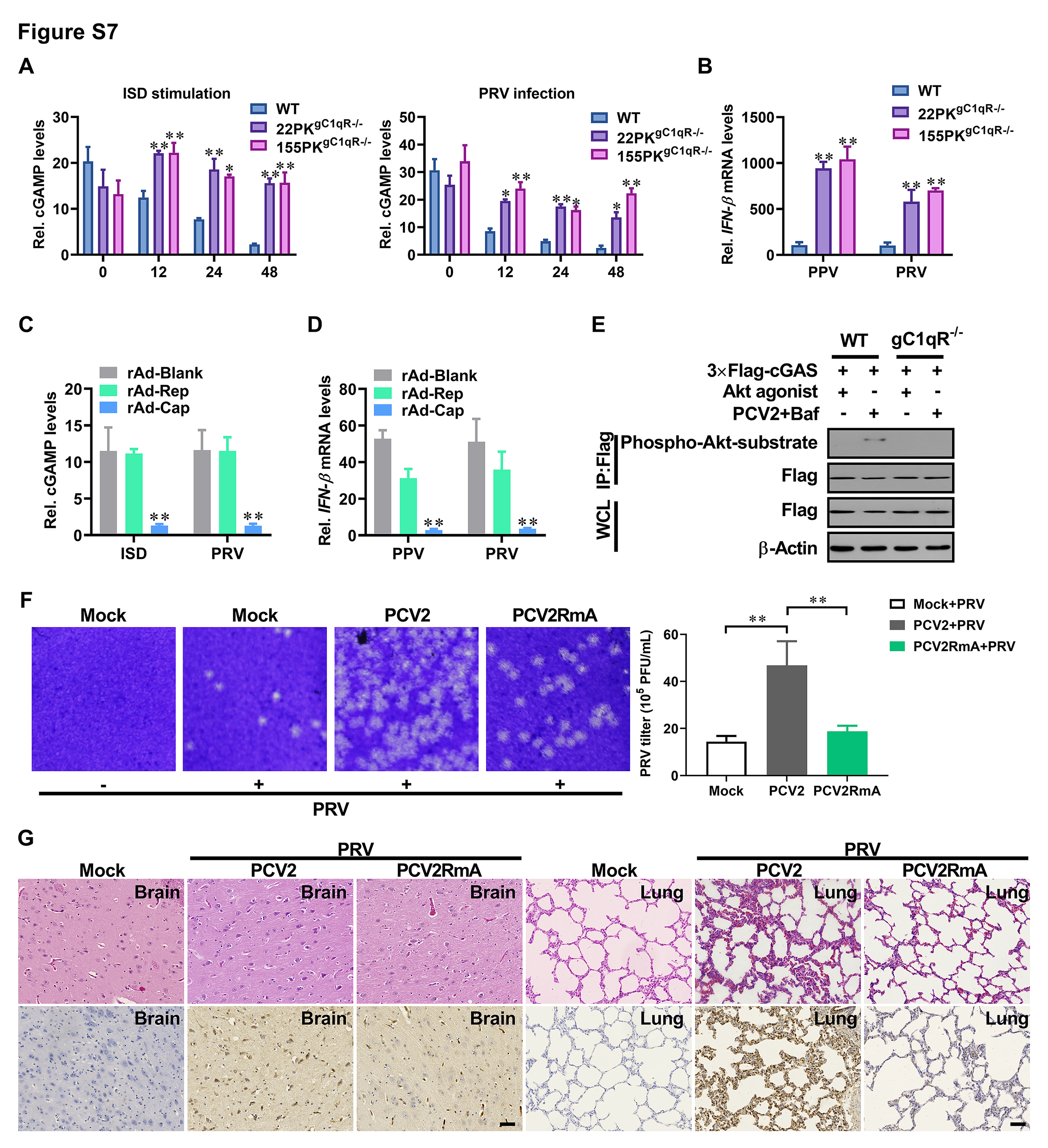

Supplement: S7 Fig — (A, B) gC1qR deficiency diminishes the inhibitory effects of PCV2 on cGAMP and IFN-β induction. gC1qR-/- PK-15 cells and wild type PK-15 cells were infected with wild type PCV2 (MOI = 5) for the indicated time, and then the relative cGAMP production levels and IFN-β mRNA levels were determined at 6 h following ISD stimulation or PPV/PRV infection. * P < 0.05, ** P < 0.01 (compared with wild-type cells). (C, D) PCV2 Cap inhibits the induction of cGAMP and IFN-β. PK-15 cells pretreated with Baf were infected with rAd-Blank (MOI = 100), rAd-Rep (MOI = 100) and rAd-Cap (MOI = 100) for 24 h, and then the relative cGAMP production levels and IFN-β mRNA levels were determined at 6 h following PPV or PRV infection by report assay (C) and qPCR respectively (D). ** P < 0.01 (compared with rAd-Blank). (E) gC1qR-/- PK-15 cells and wild type PK-15 cells were treated with Akt agonist (SC79) for 6h, then infected with Mock or PCV2 along with Baf. (F) PCV2RmA is a weak strain relative to PCV2 in promotion of DNA virus replication. PK-15 cells were infected with PCV2 (MOI = 1) or PCV2RmA (MOI = 5) for 48 h, then were further infected with PRV, and the relative viral titers were measured by standard plaque assay. Viral plaques were observed and the viral titer was calculated. * P < 0.05, ** P < 0.01. (G) PCV2RmA alleviating PRV-induced pathological changes. The piglets were infected by PCV2 (4×10^5^ TCID50), PCV2RmA (2×10^6^ TCID50) for 1 week, respectively, and then challenged with 105 TCID50 PRV for another week. Representative images of immunohistochemistry (IHC) staining for PRV using Mouse anti-gC polyclonal antibody (lower panel) and hematoxylin and eosin (H&E) staining (upper panel) in the brain and lung derived from PCV2-, PCV2RmA-, or PBS (Mock) groups challenged with PRV. Bar, 100 μm. (TIF) [file ppat.1009940.s007.tif]
