# Supplementary material for: PCV2 targets cGAS to inhibit type I interferon induction to promote other DNA virus infection
Source: PLoS Pathog. 2021 Sep 20;17(9):e1009940. doi: 10.1371/journal.ppat.1009940 (PMC8483418; doi:10.1371/journal.ppat.1009940)
Supplement: Attachment — Submitted filename: Response to Reviewers.docx [file ppat.1009940.s008.docx]

**Reviewer #1:**

**Part II – Major Issues: Key Experiments Required for Acceptance**

**1. PCV2 Cap protein was identified as the viral protein that causes cGAS phosphorylation and polyubiquitination, this was supported by the data of experiments using PCV2 bearing mutated Cap protein. To demonstrate the direct role of Cap protein, it should be tested whether expression of Cap protein alone is able to inhibit IFN-beta production through causing cGAS phosphorylation and** **polyubiquitination.**

**Response:** We appreciate all of the reviewer’s comments and suggestions. According to your suggestions, we have further examined the direct role of Cap protein in regulation of IFN-beta induction and cGAS phosphorylation and polyubiquitination and our new results showed that expression of Cap protein alone is able to inhibit IFN-beta production through causing cGAS phosphorylation and polyubiquitination. These data are presented in revised Figs. 7C-D and revised Figs. S7C-D. Description of these results are in the text, lines 368-373 and lines 415-419 of the revised manuscript. All changes or revisions are highlighted in red.

**Part III – Minor Issues: Editorial and Data Presentation Modifications**

**1. Data in Western blots need to be quantified from at least three independent experiments. The differences that were claimed in some of the Western blots are very moderate, which need to be supported by quantification.**

**Response:** We appreciate the suggestion. In response, we have quantified the Western blotting results in the revised figures and also performed statistical analyses to compare between different groups.

**2. Please indicate the source and validation of the antibody that recognizes phosphorylated S278.**

**Response:** We purchased a rabbit anti-Phospho-Akt Substrate monoclonal antibody (Cat No. #9614) which recognizes peptides and proteins containing phospho-(Ser/Thr) Akt substrate motif (RXXS*/T*) from Cell Signaling Technology. This antibody has been validated to recognize the motif (RRGS*278) of porcine cGAS. We have added this information in lines705-706 in the Materials and Methods section and in lines 1236-1237 in the Figure 5 legend in the revised manuscript.

**3. Line 33: it is not immediately clear how this study helps vaccine design.**

**Response:** We appreciate the comment. Indeed, while our series of work showing the enhancement of gC1qR-binding activity deficient PCV2 mutant (PCV2RmA), which has the potential to be developed into a vaccine, it is not immediately clear how this study helps vaccine design as alluded. We have therefore revised this sentence accordingly in lines 33-34 in the revised manuscript.

**4. What is level of IFN beta in PCV2-infected cells or piglets, without co-infection by other viruses?**

**Response:** Thanks for your suggestion. Most of studies have reported that PCV2 infection leads to immunosuppression in pigs and increases the susceptibility of infected pigs to other pathogens. Thus, we have focused on the influence of PCV2 infection on the IFN beta induced by other DNA viruses in this study. We measured the levels of IFN beta production induced by PCV2 with PCV1 as referenced in our previous studies (unpublished). IFN beta secretion can be detected in PCV1-infected cells, but we are unable to detect it in PCV2-infected cells by ELISA assay. With regard to the level of IFN beta in PCV2-infected piglets without co-infection by other viruses, we plan to measure it in the future.

**5. Line 137: if the inactivated PCV2 can also lead to cGAS degradation, does PCV2 carry enough Cap protein to target and block cGAS function?**

**Response:** Thanks for the comment and suggestion. First of all, based on results, we had observed that PCV2 can induce IFN beta production in the early phase of infection. Cap protein is the only structural protein of PCV2. Thus, we speculated that inactivated PCV2 (the main component is Cap) is able to inhibit the activation of cGAS-STING pathway. The results presented in this study confirmed that the inactivated PCV2 that carry sufficient Cap protein is able to induce cGAS reduction. We thank you for the important question.

**6. Line 424: levels of IFN beta in the media need to be directly measured by ELISA.**

**Response:** Thanks for your suggestion. We have added the data of IFN-beta in the media measured by ELISA in the revised Fig. 8K. Corresponding description of results can be found in lines 470-473 of the Result section in the revised manuscript (marked in red).

**7. Figure 1 and also in other figures: please indicate the number of piglets used in each experiment.**

**Response:** We have added the number of piglets used in each experiment in the corresponding Figure legends (Figure 1A, B, C, E, F and Figure 8L-N) or at line 727-734 in animal experiment section of the revised manuscript. Thank you.

**8. Figure 1F: what is the baseline for the quantification of IFN beta mRNA?**

**Response:** In response, we have further analyzed our data to ensure the accuracy. The QuantStudio 6 and 7 Flex Real‐Time PCR System Software can automatically calculate baseline and threshold values based on the assumption that the data exhibit a typical amplification plot. We also examined and manually adjusted the assigned baseline and threshold values according to our experiences sometimes. Thank you.

**9. Figure 3H, 4D, 6G: please present the PCV2 protein signal. Also, please quantify protein co-localization.**

**Response:** Thanks for your suggestion. We further examined the PCV2 signal in these experiments. For figure 3H, we further analyzed the signal of cGAS and PCV2 Cap protein, which is now presented in Figure S3E; for figure 4D, we further examined the signal of cGAS and PCV2 Cap protein, which is now presented in Figure S4F; for figure 6G, we further analyzed the signal of cGAS and PCV2 Cap protein, which is now presented in Figure S6G. However, since all the primary antibodies are either mouse derived or rabbit derived, it is difficult to detect the signals of three different proteins in the same experiment, so we can only present the same experiment into two parts. Meanwhile, we quantified the protein co-localization signals via analysis of the intensity profiles of indicated proteins along the plotted lines in Figure 2F, Figure 3G, Figure 4D, Figure 6G. Thank you.

**10. Figure 6D, S6E: blocking AMPK also increases cGAS level, similar to blocking Akt. Please discuss.**

**Response:** Thanks for your comments and suggestions. We have discussed the roles of AMPK and AKT signaling in regulation of cGAS level and activity at line 539-545 in Discussion section in the revised manuscript accordingly.

**11. The language needs to be edited.**

**Response:** We appreciate all of your suggestion and comments which has truly improved our manuscript. We have proofread the manuscript throughout and all the changes are highlighted in red font in the revision. Thank you.

**Reviewer #2:**

**Part II – Major Issues: Key Experiments Required for Acceptance**

**1. Major concerns:(1) Akt phosphorylates cGAS (S305, equivalent to S278 in porcine cGAS) and inhibits its catalytic activity, as referenced by the manuscript, has been reported in 2015. On the other hand, K48-linked ubiquitination of K48 has been reported to be a recognition signal for p62-dependent selective autophagic degradation (Chen et al. Mol Cell, 2016), with exactly the same lysine residue (K414 for hcGAS and K389 for porcine cGAS in this manuscript). The fact that the authors failed to cite and discuss their findings with the 2016 paper is concerning.**

**Response:** We appreciate all of your suggestions and comments. We are very sorry for our negligence of discussing this finding (Chen et al. Mol Cell, 2016) in our original manuscript. We have carefully read this paper again and now added relative discuss in lines 83-84, 571-573 and 580-588 in the Introduction and Discussion section of the revised manuscript. All of changes or revision are marked in red in the text of revised manuscript. Thank you.

**2. Firstly, the author should cite and discuss their result with the 2016 paper. Based on the 2016 paper, ISD treatment, HSV-1 infection will induce the cGAS autophagic degradation in a manner similar to the EBSS-treated condition. In this manuscript, the authors propose that HDAC6-faciliated degradation is independent of EBSS-mediated cGAS degradation. If HDAC6 recognizes K48-PolyUb, what prevents it from interacting with EBSS-mediated cGAS ubiquitination (with same Lysine)? Please provide explanation and hypothesis in the discussion.**

**Response:** Again, we apologize for not have cited the relevant literature adequately. We have added the relevant literature as new Reference 17 in the revised manuscript. As to your question on whether HDAC6 is also activated to recognizes K48-PolyUb of cGAS in EBSS-treated cells, our results showed that the deacetylase activity of HDAC6 was not markedly enhanced in EBSS-treated cells (Fig. S4E), while inhibition of the deacetylase activity of HDAC6 did not affect the interaction of ubiquitinated cGAS with p62 in EBSS-treated cells (Fig. S4J). These results indicated that EBSS-induced cGAS autophagic degradation does not depend on the activation of HDAC6. Hence, we concluded that K48-ubiquitinated cGAS are transported by activated HDAC6 in PCV2-infected cells, but K48-ubiquitinated cGAS are not specifically transported by unactivated HDAC6 in EBSS-treated cells, which lead to an increased interaction of K48-ubiquitinated cGAS with p62 in PCV2-infected cells relative to EBSS-treated cells. Based on these findings, we inferred that K48-ubiquitinated cGAS might not be prevented from interacting with HDAC6 in EBSS-treated cells, but inactivated HDAC6 is not able to transport K48-ubiquitinated cGAS. We have added the relative explanation and hypothesis in the Discussion section in lines 580-588 of the revised manuscript and marked in red font. Thank you.

**3. Figure S4B shows that HDAC6 knockdown does not increase cGAS ubiquitination in the mock control group, while Figure S4C shows it increases the basal level of cGAS-Ub in the absence of viral infection. This needs to be repeated, since the increase of cGAS-Ub upon HDAC6 KD in mock-infected group might suggest that HDAC6 is also involved in starvation-mediated cGAS ubiquitination and degradation.**

**Response:** Thanks for your comments. For Figure S4C, we repeated the experiment twice and results showed that HDAC6 knockdown does not increases the basal level of cGAS-Ub in the absence of viral infection.

**4. Figure S4D needs quantification of the cGAS signal. To me it looks like knockdown of HDAC6 prevents cGAS from being degraded within 48 hours while the author claims that HDAC6 did not affect the degradation of cGAS in EBSS-treated cells.**

**Response:** We analyzed the results of three independent experiments and further repeated this experiment once more. knockdown of HDAC6 did not appear to significantly affect the degradation of cGAS in EBSS-treated cells. To further confirm this result, we tested whether inhibition of the deacetylase activity of HDAC6 influences the degradation of cGAS in EBSS-treated cells. Results showed that inhibition of the deacetylase activity of HDAC6 did not affect the interaction of ubiquitinated cGAS with p62 and cGAS degradation in EBSS-treated cells (Fig. S4J, K). Thank you.

**5. Figure 1A-1D, details on the number of pigs infected (n=?) needs to be provided, either on the figure or in the figure legend. Please also provide details on the experiment to obtain viral loads (which tissue? qRT-PCR?) in the materials and methods for Figure 1C. For 1C and 1D, the data were presented as “relative (%)”, the authors need to clarify how data are calculated and normalized.**

**Response:** We appreciate your detailed suggestions. We have added the number of piglets used in each experiment in the corresponding figure legends (Figure 1A, B, C, E, F and Figure 8L-N) or at line 727-739 in Animal experiment section in the revised manuscript. We also revised the relative viral load into actual viral load levels in the Y axis in Figure 1C and 1D according to your suggestion. Thank you.

**6. Please provide a coIP experiment to test the affinity of WT and K389R cGAS to p62.**

**Response:** Thanks for your suggestion. We have added the coIP experiment to test the affinity of WT and K389R cGAS to p62 and presented this result in Fig. S3F in the revised manuscript.

**7. Please provide a control showing the levels of HDAC6 in Figure 4H.**

**Response:** We have moved original Fig. 4H to Fig. S4 as the revised Fig. S4H and provided a control showing the levels of HDAC6 in Fig S4H. Thank you.

**8. Figure 5H, cells reconstituted with cGAS-S278A seems to have comparable levels of cGAMP in mock and PCV2 infection. However, PCV2 should still trigger the degradation of cGAS-S278A, at least partially based on Figure 6. How does the author explain this? Please provide a WB showing the expression of these reconstituted cGAS in mock and infected group.**

**Response:** Thanks for your comments. In original Figure 5H, The cGAS^-/-^ PK-15 cells reconstituted with the WT cGAS, or cGAS mutant S278A, or cGAS mutant S278D. Before PCV2 infection, all of these cells were treated with BAF for 12 h, and then the relative cGAMP production levels were determined following ISD stimulation or PRV infection. Therefore, the degradation of cGAS was blocked by Baf, which makes the cGAS^-/-^ PK-15 cells reconstituted with cGAS-S278A seems to have comparable levels of cGAMP in mock and PCV2 infection. We are sorry for the confusion caused by our negligence in the figure legends without detailed description. We have revised the figure legend accordingly and added the WB showing the expression of these reconstituted cGAS in mock and infected group in revised Fig 5H and 5I. Thank you.

**9. Figure 6D, inhibitor of the AMPK partially saves cGAS from degradation. Could AMPK be the Akt-equivalent kinase in the EBSS-mediated cGAS degradation? Please provide thoughts on this (doesn’t need to be included in the manuscript)**

**Response:** In our study, we found that blocking of AMPK signaling can increase cGAS levels through inhibition of autophagic flux induced by PCV2, while blocking of AKT signaling can relief the suppression of cGAS activity in PCV2-infected cells, suggesting that AMPK signaling and AKT signaling regulate cGAS in different direction during PCV2 infection. Because AMPK signaling is also involved in the regulation of EBSS-induced autophagy, so AMPK signaling should be involved in the EBSS-mediated cGAS degradation. However, whether AKT signaling is activated during EBSS-mediated cGAS degradation remains to be investigated. Thank you.

**10. Figure 8B, please do a control infection with WT and RmA virus in HDAC6 knockdown cells + Akt inhibitor, and monitor IFN-b mRNA levels, to test whether the effect of the elevated IFN-b is solely due to the rescue of cGAS level and activity.**

**Response:** Thanks for your suggestion. We further tested whether simultaneous rescue cGAS level and activity through knockdown of HDAC6 and inhibition of AKT signaling could completely remove the inhibitory effects of PCV2 on IFN-β induction. Results showed that PPV or PRV-induced IFN-β expression did increase a litter bit in PCV2-infected cells when HDAC6 and AKT were simultaneously silenced or inhibited, but IFN-β expression was still lower in PCV2-infected HDAC6 knockdown cells than that in PCV2RmA-infected HDAC6 knockdown cells in the presence of AKT inhibitor (LY294002) (Fig. 8B). However, cGAMP production was markedly increased in PCV2-infected cells when HDAC6 and AKT were simultaneously silenced or inhibited, and showed a similar level between PCV2-infected HDAC6 knockdown cells and PCV2RmA-infected HDAC6 knockdown cells in the presence of AKT inhibitor (LY294002) (Fig. 8C). These data suggested that simultaneous rescuing cGAS level and activity through knockdown of HDAC6 and inhibition of AKT signaling indeed remove the inhibitory effects of PCV2 on cGAMP production, but cannot effectively remove the inhibitory effects of PCV2 on IFN-β induction. Notably, PCV2RmA, a gC1qR binding activity deficient PCV2 mutant, did not exhibit significant inhibitory effect on cGAMP and IFN-β production induced by ISD stimulation, PRV or PPV infection, whenever HDAC6 and AKT were silenced/inhibited or not (Fig. 8A, B, C). We have added the relative results at line 429-446 in Results section and relative figures in Figure 8 B and C in the revised manuscript and marked in red font. Thank you.

**11. Provide a mock-infected Brain and Lung IHC for Figure S7.**

**Response:** Thanks for your suggestion, and we have now provided a mock-infected Brain and Lung IHC in revised Figure S7E.

**Part III – Minor Issues: Editorial and Data Presentation Modifications**

**(1) Define “EBSS” in the abstract (Line 24)**

**Response:** Thanks for your suggestion. We have revised the abstract and defined the “EBSS” at line 24-25 in the Abstract.

**(2) Please provide background on cGAS Polyubiquitination studies before directly mentioning the ‘spatially transportation of ….” (Line 81)**

**Response:** We have added this part at line 79-87 in the Introduction section in the revised manuscript. Thank you.

**(3) Provide more background on PCV2 in the introduction part, (e.g., DNA or RNA virus, size, life cycle, etc.)**

**Response:** We have added the background of PCV2 at line 93-101 in the Introduction section in the revised manuscript. Thank you.

**(4) Again define “EBSS” in the result part (Line 178)**

**Response:** We have defined the “EBSS” at line 189-191 in the Result section in the revised manuscript. Thank you.

**(5) Line 183, “although” should be deleted.**

**Response:** We are sorry for this mistake. We have corrected the line 196 in the revised manuscript. Thank you.

**(6) Line 187, how do you narrow down the region to only include 8 Ub sites? More details need to be given to justify the logic here.**

**Response:** Thanks for your suggestion. For the prediction of the potential ubiquitination sites in porcine cGAS, we followed the method reported in previous study (Xu. et.al, Journal of genetics and genomics, 2017). Briefly, we firstly analyzed the conserved regions of cGAS between homo sapiens and sus scrofa (residues 135-495 in porcine align to the residues 161-522 in humans). Next, we analyzed the possible lysine modification in human cGAS (Uniprot Accession number: Q8N884) through a predictor of ubiquitination sites, PLMD (Protein Lysine Modification Database, <http://plmd.biocuckoo.org/index.php>). Lastly, we predicted the potential ubiquitination sites in porcine cGAS through alignment of similar conserved sites between human cGAS and porcine cGAS. We have added a brief description for this part at line 199-201 in the Results section of the revised manuscript. Thank you.

**(7) Line 201-205 needs revision. I understand that the author wants to propose a different model for PCV2-mediated cGAS autophagy. However, the K48 Ub and the site is shared between the EBSS and PCV2. In this case, the author cannot claim that K48 Ub chains serves as a signal only for the selective autophagy by PCV2 (Line 205).**

**Response:** We very appreciate this important comment. We have rewritten this part at line 217-221 in the begin of Result 4 of the revised manuscript. Thank you.

**(8) Line 416, why is Figure S7C described here? The whole section is on PCV2RmA mutant virus.**

**Response:** Thanks for your suggestion. We have rearranged the layout of the results according your suggestion. Original Fig. S7C was adjusted into revised Fig. S7E, and corresponding result description were presented at line 420-423 in the begin of second paragraph in the Result 8 section of the revised manuscript and marked in red font. Thank you.

**Reviewer #3:**

**Part II – Major Issues: Key Experiments Required for Acceptance**

**1. Line 81-87. This part is somewhat over-exaggerated. The findings in this manuscript are based on PCV infection, which may not be generalized.**

**Response:** We very much appreciate all of your suggestions and comments, which has significantly improved our paper. In response, we have re-written this part in lines 87-92 in the revised manuscript and marked in red font. Thank you.

**2. Figure 3D, cGAS was still degraded with Baf-A1 treatment, why?**

**Response:** Thanks for your comments. In Figure 3D, PK-15 cells were treated with EBSS together with or without PCV2 infection (MOI=5) in the presence of BAF. The poly-ubiquitination levels were increased, while the protein levels of cGAS were decreased when cells were treated with EBSS or infected with PCV2, or both EBSS and PCV2. Initially, we could not understand why cGAS was still degraded in the presence of Baf-A1 in doing these kinds of experiments. By analyzing other studies, we now know that Ubiquitin is a small protein of 76 amino acids that can be covalently linked to targeted proteins to form into poly-ubiquitinated targeted proteins, leading to the molecular weight change of poly-ubiquitinated targeted proteins. Thus, un-ubiquitinated targeted proteins will be decreased in cells in western blotting detection, when part of targeted proteins were poly-ubiquitinated. BAF treatment can block the degradation of poly-ubiquitinated targeted proteins, resulting in the accumulation of poly-ubiquitinated targeted proteins, and further slowdown the poly-ubiquitination of targeted proteins, but un-ubiquitinated targeted proteins were also decreased due to the poly-ubiquitination of targeted proteins. Thank you.

**3. Line 183, ‘although’ typo.**

**Response:** We are sorry for this mistake and have corrected at line 196 in the revised manuscript. Thank you.

**4. Line 225, the interpretation for fig 4E is not very accurate. K389R mutant was still ubiquitinated but it did not interact with HDAC6.**

**Response:** We very much appreciated your suggestion and have corrected this sentence in line 241-243 in the revised manuscript.

**5. Figure 4F and G, the immunoprecipitated bands of p62 are kind of dubious.**

**Response:** We replaced the results of another independent experiment in the revised Figure F and G in the revised manuscript. Thank you.

**6. Regarding p62 recruitment, previous study reveals that K414 ubiquitination of cGAS helps to recruit p62 (PMID: 27666593), which is contradictory to current study. Authors may consider providing some experimental evidence or discussion at least.**

**Response:** We appreciate for your suggestion. Previous study revealed that ubiquitination of cGAS helps to recruit p62 to promote cGAS degradation (PMID: 27666593). In this study, we firstly predicted the potential ubiquitination sites in porcine cGAS through alignment of similar conserved sites between human cGAS and porcine cGAS (Fig. S3B), and mapped potential ubiquitination sites in porcine cGAS. Different from the lysine residue at position 414 as a key residue for K48-linked poly-ubiquitination of human cGAS, the lysine residue at position 389 of porcine cGAS is required for its K48-linked poly-ubiquitination regardless of PCV2 infection or EBSS treatment, which was evidenced by the data that the K48-linked ubiquitination of K389R cGAS mutant was abrogated in both PCV2-infected cells and EBSS-treated cells (Fig. 3E, F, Fig. S3C). PCV2 infection also promoted the interaction of wild-type cGAS with p62 but failed to promote the interaction of K389R porcine cGAS mutants with p62 (Fig. 3G, H, Fig. S3E, S3F). In the original manuscript, we did not summarize and discuss this part, which we apologize. We have added this part in lines 589-601 in the Discussion section of the revised manuscript.

**7. Figure 4H and I, it is not very convincing to conclude the necessity of deacetylase activity solely depending on an inhibitor, considering the potential off-target effect.**

**Response:** We appreciate your detailed suggestion. To further confirm this finding, we examined the interaction of ubiquitinated cGAS with p62 and cGAS levels in the HDAC6 deficient cells that rescued with mutated porcine HDAC6 (D538A/D608A/D609A, named as HDAC6-3M), which abolished the deacetylation activity of HDAC6 in these cells, while significantly decreased the interaction of ubiquitinated cGAS with p62 and the degradation of cGAS during PCV2 infection. We have added these results as the revised Figure 4H and 4I, and moved the original Fig. 4H, 4I to Fig. S4 as the revised Fig. S4H and S4I. The related results were described in line 254-259 in the revised manuscript. Thank you.

**8. Figure5C and D, it seems that the production of cGAMP was not affected by PCV, why?**

**Response:** Thanks again for your comments. In Figure 5C, the cGAS^-/-^ PK-15 cells were reconstituted with the wild type cGAS, mutant cGAS (K389R), respectively, then infected with PCV2 (MOI=5) for 48 h to detect the relative cGAMP production levels at 6 h following ISD stimulation or PRV infection. In Figure 5D, PK-15 cells pretreated with TSA (DMSO as control) were infected with PCV2 (MOI=5) for 48 h, then the relative cGAMP production levels were determined at 6 h following ISD stimulation or PRV infection. All the cells were infected PCV2 for to detection of the relative cGAMP production levels at 6 h following ISD stimulation or PRV infection. If mutation of K389 site of porcine cGAS and inhibition of HDAC6 activity to prevent the degradation of K48-ubiquitinated cGAS were not sufficient to weaken the inhibitory effects of PCV2 on cGAMP induction, there it would be no significant changes in cGAMP in PCV2 infected these cells. The results presented here confirmed that the block of cGAS autophagic degradation is unable to restore the function of cGAS. production of cGAMP was still affected by PCV2, and preventing the degradation of K48-ubiquitinated cGAS were not able restore the function of cGAS in PCV2-infected cells. Thanks.

**9. For figure 5 and 6, the authors heavily relied on an Akt inhibitor and a p-substrate antibody. It would be more convincing to provide some genetic evidence.**

**Response:** Thanks for this important comment. In our study, we found PCV2 promotes the phosphorylation of cGAS at S278 by western blotting using a rabbit anti-Phospho-Akt Substrate monoclonal antibody that has been validated to be able to recognize the motif (RRGS*278) of porcine cGAS. To further confirm the role of S278 phosphorylation in the modulation of porcine cGAS activity, we also compared the enzyme activities of wild-type porcine cGAS, phosphorylation-resistant mutant S278A, and phosphomimetic S278D mutant via *in vitro* catalytic activity assay. Results showed that purified cGAS S278A mutant proteins possessed a similar cGAMP production activity as wild-type cGAS *in vitro* (Fig. 5G), whereas the same dose of phosphomimetic S278D mutant was not able to catalyze the substrate to produce cGAMP in the same reaction condition (Fig 5G and S5B, C). To investigate which signaling was responsible for the phosphorylation of porcine cGAS at S278 during PCV2 infection, we used related inhibitors and specific siRNAs to examine the potential roles of PI3K/Akt, p38-MAPK, JNK, ERK and AMPK signaling pathways in the phosphorylation of porcine cGAS and regulation cGAMP production (Fig.6A, B and Fig. S6A, B). In addition, we used cGAS^-/-^ PK-15 cells were reconstituted with the WT cGAS, or S278A mutant, or S278D mutant, then infected with PCV2 to detect the poly-ubiquitination levels and phosphorylation levels of cGAS in these cells. Result showed that the phosphorylation of cGAS at S278 is not required for K48-linked poly-ubiquitination and degradation of porcine cGAS, but the phosphorylation of porcine cGAS at S278 indeed can facilitate the K48-linked poly-ubiquitination of porcine cGAS during PCV2 infection. Thank you.

**10. Figure 7, what is the role of gC1qR in cGAS activation in general? How is gC1qR relayed to AKT and PKCδ? Similarly, it would be more convincing if authors could provide some genetic evidence other than relying on a PKCδ inhibitor.**

**Response:** In this study, we demonstrated that gC1qR mediated PCV2-induced PI3K/Akt activation to inhibit cGAS enzymatic activity directly. In our previous study, we have confirmed that PCV2 infection could activate PI3K/Akt signaling pathways through the mediation of PCV2 Cap binding protein gC1qR, and found that PKC-δ was phosphorylated by PLC-mediated signaling in PCV2 infection, and gC1qR functioned as an adaptor to recruit phosphorylated PKC-δ (Wang et. al, Journal of Virology 2019, PMID: 31511386). Thus, we did not provide these data in this study. According to your suggestion, we detected the Ac-tubulin levels in the PKC-δ knockdown cells. knockdown of PKCδ also could suppress HDAC6 activation in either PCV2-infected cells or the Cap-expressing cells. We have added these results as revised Figure 7J and 7L. The relative results were described in line 398-406 of the revised manuscript. Thank you.

**11. For figure 8, it would be critical to show that the mutant virus didn’t recruit gC1qR and gC1qR deficiency could abrogate the immune modulatory effect of PCV infection.**

**Response:** Thanks for your comments. Our previous study showed that N-terminal 24RRR26 of PCV2 Cap, a highly conserved motif among circovirus species, was required for interacting with gC1qR, and that mutation of this motif impeded PCV2 Cap recruit gC1qR, and PCV2RmA mutants didn’t recruit gC1qR. gC1qR deficiency could abrogate immune modulatory effect of PCV2 infection in regulation of macrophage function and Th1 immune. These data have been published in two articles (Wang et. al, Journal of Virology 2019, PMID: 31511386; Du et al, Journal of Immunology 2018, PMID: 29858268). Thank you.

**12. The discussion part needs to be significantly improved. The logical flow between paragraphs are vague.**

**Response:** We appreciate all of your suggestion and comments, which has helped us to improve our manuscript. We have significantly revised the Discussion part and other parts of our revised manuscript, and all the changes were highlighted in red font in the revision. Thank you.
