# Supplementary material for: PCV2 targets cGAS to inhibit type I interferon induction to promote other DNA virus infection
Source: PLoS Pathog. 2021 Sep 20;17(9):e1009940. doi: 10.1371/journal.ppat.1009940 (PMC8483418; doi:10.1371/journal.ppat.1009940)
Supplement: Attachment — Submitted filename: Response to Reviewers.docx [file ppat.1009940.s009.docx]

**Reviewer #2:**

**Part I - Summary**

**1. Some minor suggestions: Line 580-588. Please edit the language to make it concise and accurate. For example, “PCV2 infection, but not EBSS treatment, activates HDAC6 to facilitate cGAS transportation, leading to an increased interaction between K48-ubiquitinated cGAS with p62”. “Inactivated HDAC6 may interact with K48-Ub cGAS but is not able to transport it.”**

**Response:** We very appreciate your suggestion. We have rewritten this part at line 585-589 in the Discussion of the revised manuscript. All changes or revisions are highlighted in red. Thank you.

**Reviewer #3:**

**Part I - Summary**

**1. Line 73. Reference #9 is incorrect, and the sentence needs to be rephrased. PMID: 34015248 and PMID: 30092200 should be referenced.**

**Response:** We appreciate all of the reviewer’s comments and suggestions. According to your suggestions, we have deleted Reference #9 and added the relevant literature as new Reference #9, and #12 in the revised manuscript. Rephrased description can be found in lines 71-75, 77-78 in the revised manuscript (marked in red). Thank you.

**2. The writing still needs to be improved. For instance, line 83 ‘through RNF185 mediation’; line 87 ‘despite some molecules that’; line 91-92, grammar; line 99, deplete ‘as most people known’. The list is by no means exhaustive.**

**Response:** We very much appreciate all of your suggestions and comments. According to your suggestions, line 84-85, the statements of “through RNF185 mediation” were corrected as “K27-linked polyubiquitination of cGAS at Lys173and Lys384 by RNF185 promotes its enzymatic activity during HSV-1 infection”; line 90-91, the statements of “despite some molecules that” were corrected as “several molecules have been identified that regulate the enzymatic activity and degradation of cGAS”; line 91-95, the statements of “For example, it is unclear whether some specific molecules participate spatially transportation of the ploy-ubiquitinated cGAS from cytosol to the autolysosome; whether other types of post-translation modifications of cGAS are associated with the polyubiquitination of cGAS, all of these question remain to be answered” were corrected as “For example, it is unclear whether some specific molecules participate in spatially transportation of the ploy-ubiquitinated cGAS from cytosol to the autolysosome and whether other types of post-translation modifications of cGAS are associated in the polyubiquitination of cGAS. All of the above-mentioned questions remain to be answered.”; line 102, “as most people known” was deleted. We tried our best to improve the manuscript and made some changes in the manuscript. These changes will not influence the content and framework of the paper. And here we did not list the changes but marked in red in revised manuscript. Once again, thank you very much for your comments and suggestions.

**3. Line 247, the reconstitution experiments with HDAC6 WT and mutant were not described.**

**Response:** We appreciate your detailed suggestions. We have added the relevant description of reconstitution experiments with HDAC6 WT and mutant at line 248-251 in Result 4 section in the revised manuscript. Thank you.
